# Bloodstream Infection Due to Coagulase-Negative Staphylococci: Impact of Species on Prevalence of Infective Endocarditis

**DOI:** 10.3390/antibiotics12091453

**Published:** 2023-09-18

**Authors:** Sara F. Haddad, Brian D. Lahr, Sebastian Santos Patarroyo, Supavit Chesdachai, Kami D. Kies, John C. O’Horo, Daniel C. DeSimone, Parham Sendi, Larry M. Baddour

**Affiliations:** 1 Division of Public Health, Infectious Diseases and Occupational Medicine, Department of Medicine, Mayo Clinic College of Medicine and Science, Mayo Clinic, Rochester, MN 55905, USA; santospatarroyo.sebastian@mayo.edu (S.S.P.);; 2 Division of Clinical Trials and Biostatistics, Mayo Clinic College of Medicine and Science, Mayo Clinic, Rochester, MN 55905, USA; 3 Clinical Microbiology Core Laboratory, Division of Clinical Microbiology, Department of Laboratory Medicine and Pathology, Mayo Clinic College of Medicine and Science, Mayo Clinic, Rochester, MN 55905, USA; kies.kami@mayo.edu; 4 Division of Pulmonary and Critical Care, Department of Medicine, Mayo Clinic College of Medicine and Science, Mayo Clinic, Rochester, MN 55905, USA; 5 Department of Cardiovascular Medicine, Mayo Clinic College of Medicine and Science, Mayo Clinic, Rochester, MN 55905, USA; 6 Institute for Infectious Diseases, University of Bern, 3001 Bern, Switzerland

**Keywords:** bacteremia, bloodstream infection, coagulase-negative staphylococci, species identification, infective endocarditis

## Abstract

(1) Background: Coagulase-negative staphylococci (CoNS) are an important group of organisms that can cause bloodstream infection (BSI) and infective endocarditis (IE). The prevalence of IE in patients with BSI due to different CoNS species, however, has received limited attention; (2) Methods: A retrospective study of adults with monomicrobial CoNS BSI who had undergone echocardiography and a risk factor analysis was done to determine the most common CoNS species that cause definite IE; (3) Results: 247 patients with CoNS BSI were included in the investigation; 49 (19.8%) had definite IE, 124 (50.2%) possible IE, and 74 (30.0%) BSI only. The latter two entities were grouped in one category for further analysis. The most common species in CoNS BSI was *Staphylococcus epidermidis* (79.4%) and most patients (83.2%) had possible IE/BSI only. 59.1% of patients with BSI due to *S. lugdunensis* had definite IE. The majority of CoNS were healthcare-associated/nosocomial bacteremia. Multivariable analysis demonstrated that valve disease (*p* = 0.002) and a foreign cardiovascular material (*p* < 0.001) were risk factors associated with definite IE. Patients with *S. lugdunensis* BSI had an 8-fold higher risk of definite IE than did those with *S. epidermidis* BSI and nearly a 13-fold higher risk than did patients with BSI due to other species of CoNS (*p* = 0.002); (4) Conclusions: The prevalence of definite IE in patients with BSI due to different CoNS species was significant. CoNS bacteremia, particularly with *S. lugdunensis*, confers a significant risk of IE, particularly in patients with a valve disease or intravascular foreign body material and should not be immediately dismissed as a contaminant.

## 1. Introduction

Coagulase-negative staphylococci (CoNS) are a varied group of organisms that can cause bloodstream infection (BSI) and other types of infections. The source of CoNS BSI is commonly but not always associated with infection of an indwelling medical device. With an increasing number of prosthetic devices being implanted and an aging population globally, the role of CoNS needs re-evaluation, and an appreciation of CoNS species identification in infective endocarditis (IE) is warranted. Recent advances in microbiologic screening have identified new species and dissected apart the larger group of CoNS and allowed for new insights about species and subtypes that wasn’t previously feasible such as recognizing the role of different species of CoNS in causing both native tissue and prosthesis-related infections [1].

The primary aims of this investigation were to characterize the species distribution in patients with CoNS BSI and evaluate risk factors associated with the development of definite IE in this setting.

## 2. Results

**Figure 1:**
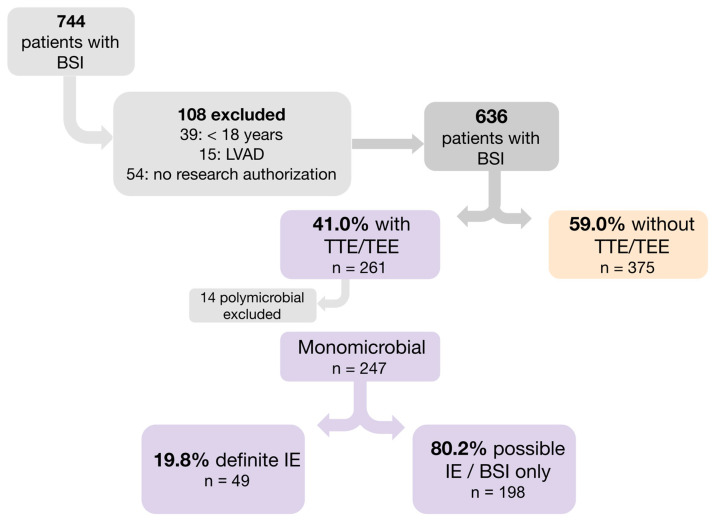
Flow Chart of Patients Identified for Study Inclusion. BSI, bloodstream infection; IE, infective endocarditis; TEE, transesophageal echocardiogram; TTE, transthoracic echocardiogram.

Overall, 744 patients with initial episodes of CoNS BSI were identified and 108 of them did not meet inclusion criteria (Figure 1). Among the remaining 636 patients, 261 (41.0%) had undergone echocardiography; 14 patients who had polymicrobial BSI were excluded. The remaining 247 patients with monomicrobial CoNS BSI in whom echocardiography was done comprised the study population.

Of the 247 patients included in subsequent analysis, the majority were male (66.8%) and white (90.7%), with a median age of 64.6 (IQR 53.8–72.6) years at the time of BSI. Based on modified Duke criteria, 49 (19.8%) patients were classified as definite IE, 124 (50.2%) as possible IE, and 74 (30.0%) as BSI only. Compared to patients with possible IE/BSI only, patients with definite IE were older (median age 69.5 [IQR 59.7–75.7] vs. 62.8 [IQR 53.0–70.8], *p* = 0.017) and were more likely to have chronic heart failure (53.1% vs. 24.2%, *p* < 0.001) (Table 1). In patients with definite IE, healthcare-associated BSI was more frequent than in those with possible IE/BSI (61.2% vs. 47.0%, *p* < 0.001), whereas in patients with possible IE/BSI only nosocomial infections were more frequent than in those with definite IE (42.9% vs. 14.3%, *p* < 0.001). 29.6% of the patients were receiving immunosuppressive therapy. Patients with definite IE had a higher prevalence of congenital or acquired valvular disease (65.3% vs. 25.8% *p* < 0.001), prosthetic valves (40.8% vs. 10.6%, *p* < 0.001), CIED (46.9% vs. 16.7%, *p* < 0.001), and prosthetic joints (26.5% vs. 10.1%, *p* = 0.002) as compared to that of the possible IE/BSI only group. Conversely, patients with possible IE/BSI only were more likely to have a central or peripherally inserted central catheter (54.5% vs. 32.7%, *p* = 0.009) (Table 1).

**Figure 2:**
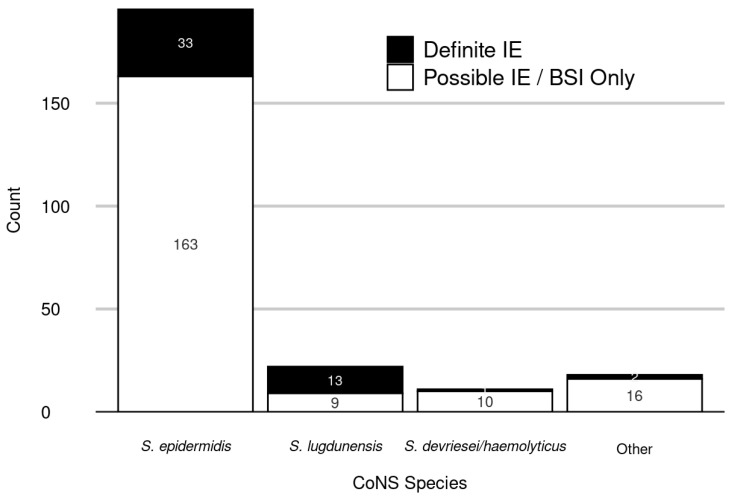
CoNS Species Distribution Among Patients with Definite IE and Possible IE/BSI only.

Figure 2 displays the distribution of CoNS species identified in the 198 patients with possible IE/BSI only and the 49 patients with definite IE. *S. epidermidis* accounted for the large majority (196/247, 79.4%) of detected species and was the predominant species in both possible IE/BSI only (163/198, 82.3%) and definite IE cases (33/49, 67.3%). Of note, *S. lugdunensis* was identified in 26.5% (13/49) of patients with definite IE compared to only 4.5% (9/198) in patients with possible IE/BSI only. Patients with possible IE/BSI only had a shorter TTP (median 19 [IQR 15–22] vs. 21 [IQR 16–24] hours, *p* = 0.035). Methicillin resistance was identified in CoNS BSI isolates in 52.1% of definite IE patients and 71.0% of possible IE/BSI only patients (*p* = 0.011) (Table 2).

Among the 49 cases of definite IE, the source of infection was undefined in 41 (83.7%); 4 of 8 cases with an identified source of infection were due to skin or soft tissue infections. The primary imaging modality was echocardiography, with transesophageal echocardiogram (TEE) the most used (98.0%) procedure, followed by transthoracic echocardiogram (TTE) (73.5%) and positron emission tomography–computed tomography (24.5%). In this subgroup, 23 patients (46.9%) had a CIED, although only 15 (30.6%) had CIED IE. The aortic valve was affected in 44.9% of patients, while the mitral, tricuspid, and pulmonary valves were affected in 22.4%, 8.2%, and 2.0%, of them, respectively.

Cardiac valve tissue was sent for culture in 9 of 17 patients who underwent cardiac surgery, with 7 positive cultures, and implantable devices were cultured in 11 patients, of whom 8 had a positive culture. Methicillin resistance was reported in 52.1% of CoNS isolates from patients with definite IE.

Patients with definite IE had a median length of hospital stay of 15 days (IQR 11–26), and 18 (36.7%) patients were admitted to the ICU for a median length of stay of 7 days (IQR 2–11). In-hospital mortality was 18.4%, and cumulative mortality at 5-year follow-up was 54.5%. Overall, 63.3% of patients had at least one complication, with the most common complications being perivalvular abscess (28.6%), central nervous system emboli (22.4%), musculoskeletal infection (18.4%), and new development of valvular insufficiency (14.3%). Valve surgery was done in 17 (34.7%) patients. Patients with native valve endocarditis (NVE) had high rates of morbidity and mortality, with 60.9% developing complications and a 30.4% in-hospital mortality.

Multivariable analysis showed that native valve disease (odds ratio [OR] 3.24, 95% confidence interval [CI] 1.54–6.80, *p* = 0.002) and the presence of an intravascular foreign device (either CIED or prosthetic valve; OR 6.28, 95% CI 2.98–13.26, *p* < 0.001) were both significantly associated with increased risk of definite IE. Other variables in the model, namely age, CCI, and hemodialysis, did not have a statistically significant association with risk of definite IE.

In the secondary multivariable analysis, CoNS species was identified as a significant risk factor associated with definite IE (*p* = 0.002). Patients with *S. lugdunensis* isolated from blood cultures were 8-times more likely to have definite IE as compared to those with *S. epidermidis* (OR 8.02, 95% CI 2.40–26.90) and nearly 13-times more likely than those with other species (OR 12.89, 95% CI 2.34–70.90) (Table 3).

## 3. Material and Methods

### 3.1. Study Design and Participant

A retrospective cohort study of patients ≥ 18 years admitted to Mayo Clinic Rochester between January 2016 and July 2022 was conducted. Patients who had opted out of research participation were excluded. Patients with CoNS BSI were identified in a Mayo Clinic clinical laboratory database. Patient-related variables were then evaluated and extracted from the electronic health record (EHR).

Subjects who had ≥2 positive blood cultures for CoNS with the same species of CoNS and the same phenotypic antimicrobial susceptibility pattern in time window of a 48 h were included. Only the initial episode of CoNS BSI was included and polymi-crobial BSI were excluded. Only those patients were included in the analysis in whom an echocardiographic examination was performed ≤1 week prior to and ≤4 weeks after the first positive blood culture. Patients with a left ventricular assist device were ex-cluded because defining IE in this population is difficult. The echocardiographic find-ings and the diagnosis of IE were categorized according to the modified Duke criteria [2]. Patients categorized with definite IE were included in a “definite IE” group, whereas patients classified with possible or rejected IE were included in a “possible IE/BSI only” group.

### 3.2. Microbiologic Methods

Our medical center utilized Becton Dickinson (BD) BACTEC FX (Becton-Dickinson, Sparks, MD, USA, 1990) instruments to detect blood culture positivity during the study period. When a blood culture flagged positive, a Gram stain was performed, and the bottle was subcultured. Species identification was done on newly recovered isolates which underwent matrix-assisted laser desorption/ionization-time of flight mass spectrometry (MALDI-TOF MS) MALDI Biotyper^®^—Bruker (Salt Lake City, UT, USA) unless they fit the “same morphology rule”. This rule applied to cultures that were positive/growing the same isolate morphology and the culture/collect date was the same day, consecutive or every other day. For example, if a patient had blood cultures collected on day 1 with staphylococci, then the BioFire FilmArray panel BCID2 (bioMérieux SA, Salt Lake City, UT, USA) would be tested with the MALDI-TOF. If a second draw that was collected on the same patient on day 2 and flagged positive with the same morphology as that from day 1 collection, then BioFire and not MALDI-TOF would be performed. If MALDI-TOF did not provide a genus/species level identification, then latex agglutination was used. Time to positivity (TTP), defined as the time between the start of incubation and the start of the alert signal, was recorded for each bottle from the initial blood culture series. When both aerobic and anaerobic bottles were positive, the shortest TTP was used.

### 3.3. Definitions

The following definitions were used to classify cases:

- CoNS BSI episode: Only a patient’s initial CoNS BSI episode during the study period was included if they had at least two or more positive blood cultures for the same CoNS species within 48 h.
- Community-onset BSI: positive blood culture collected from patients at the time of hospital admission or within 48 h of admission.
- Health care-associated BSI: BSI that occurred in patients with prior healthcare exposure such as intravenous therapy or chemotherapy, wound care, hemodialysis, or a specialized nursing care in the 30 days before developing BSI, a hospitalization for two or more days in the 3 months preceding the BSI, or a residence in a nursing home or long-term care facility.
- Nosocomial BSI: A positive blood culture acquired after two days of hospitalization [3].
- Cardiovascular implantable electronic device (CIED): included pacemaker, implantable cardioverter defibrillator, cardiac resynchronization therapy device.
- Definite CIED-IE: clinical evidence of a pocket or generator infection, with two major criteria, or one major criterion plus three minor criteria [4].
- Immunosuppressive therapy: patients receiving immunosuppressive treatment ≤ 2 months after solid organ transplantation, patients receiving daily corticosteroid therapy with a dose ≥ 20 mg of prednisone or equivalent for ≥14 consecutive days, and patients receiving biologic immune modulators [5]. HIV and cancer (solid organ and hematological) were collected as separate variables.
- Native, congenital or acquired valvular disease: moderate to severe stenosis, regurgitation, or atresia of any valve.
- Charlson comorbidity index (CCI): comorbid conditions incorporated in CCI were not chart-abstracted like the other clinical data, rather the patients’ ICD9/10 codes were extracted from their electronic medical record and used to define presence/absence of each condition [6].

### 3.4. Objective

The primary objective was to determine risk factors associated with developing IE in subjects with CoNS BSI. Secondary objectives were to characterize the distribution of CoNS species and assess the impact of species on prevalence of IE.

### 3.5. Statistical Analysis

Descriptive statistics were expressed by median with interquartile range (IQR) for continuous variables and by frequency and percentage for categorical variables. Unadjusted analyses via Wilcoxon rank sum and Pearson χ^2^ tests for continuous and categorical variables, respectively, were used to compare baseline characteristics for the patients with definite IE versus possible IE/BSI only. Multivariable analysis based on binary logistic regression was used to model the relationship between multiple covariates and the risk of definite IE. To avoid overfitting, we limited the number of terms in the model by adhering to a widely used rule of thumb requiring at least 10 cases per candidate risk factor. The following covariates were prespecified to account for previously demonstrated risk factors of IE: age [7], CCI [8], hemodialysis [8], valve disease [9], and foreign device [5]. A second multivariable logistic regression analysis was conducted to explore the risk of definite IE from CoNS species (*S. epidermidis*, *S. lugdunensis*, other) after controlling for the same covariates above. However, due to the effective sample size constraints, we reduced the covariates in this model by combining the effects for age and CCI into a single variable using the age weighted CCI score. For both models, age and/or CCI were initially modeled with splines to evaluate the significance of their nonlinear effects; because these tests were nonsignificant, the final models were re-fit using linear terms for both variables. Statistical significance was defined by *p* < 0.05. All analyses were performed using the statistical programming language R, version 4.2.2 (R Foundation for Statistical Computing, Vienna, Austria) [10,11,12,13].

## 4. Discussion

The current investigation highlights the importance of species designation of CoNS BSI isolates and its importance in estimating its risk for complicating definite IE. *S. epidermidis* was the most common species identified in both possible IE/BSI only and definite IE cases. However, the proportion of definite IE was highest (59.1%) for *S. lugdunensis*; this contrasted with the prevalence due to *S. epidermidis* (16.8%), *S. devriesei/haemolyticus* (9.09%) and other (11.1%) CoNS species. In line with these observations, multivariable analysis demonstrated that the odds for IE were almost 13 times higher after *S. lugdunensis* BSI in comparison to that of all other CoNS BSI, and 8 times higher in comparison to that after *S. epidermidis* BSI.

The remarkably high (59.1%) prevalence of IE observed in patients with *S. lugdunensis* BSI within our study is indeed noteworthy. Interestingly, rates of IE among patients with BSI due to *S. aureus* have ranged from 6% to 32% in prior reports [14]. Our findings underscore the critical importance of recognizing *S. lugdunensis* as a significant pathogen capable of causing IE, which was highlighted in the 2023 Duke-ISCVID IE updated diagnostic criteria that, for the first time listed *S. lugdunensis* as a “typical pathogen” to cause IE [15]. Only one other retrospective investigation has been published that evaluated the impact of CoNS species on prevalence of IE. When compared to the prevalence (2%) of IE due to *S. epidermidis*, the IE prevalence was higher (14%, *p* < 0.01) for BSI cases due to *S. lugdunensis* [16]. Moreover, prevalence of IE and other outcomes were like that in patients with BSI due to *S. aureus*, which supports the tenet that the species *lugdunensis*, like *aureus*, are formable pathogens. These findings are consistent with the general impression that *S. lugdunensis* is a unique species in its ability to cause IE.

The prevalence of definite IE among all CoNS BSI was 19.8% in our study. This is considerably higher than that (7.7%) described among patients with BSI at two Danish hospitals [17]. It is conceivable that the lower prevalence in the Danish investigation was impacted by whether echocardiography was done or not. Although no CoNS- specific data were available related to whether echocardiography was performed, there were data for all Gram-positive cocci that caused BSI in the study; of note, BSI due to CoNS was seen in only 11.1% (65/585) of the overall study cohort. For this cohort, 12.3% of BSI patients had IE; the rate (17.4%) increased by almost one-third in the subset of patients who underwent echocardiography.

In a nationwide investigation from Denmark [18], the prevalence of IE in patients with CoNS bacteremia was 1.6% if only one set of blood cultures was considered. The IE rate increased to 8.1% when two blood cultures sets were included. A diagnostic evaluation that included echocardiography for IE, however, was not systematically performed and prompted the investigators to warn that their findings should be interpreted as conservative estimates [18]. Thus, the results of our study suggest that the prevalence of IE in the setting of CoNS BSI may be higher than previously reported when at least two sets of blood cultures (i.e., continuous bacteremia) are considered with a systematic echocardiographic investigation, particularly in BSI cases due to *S. lugdunensis*. The lack of such an investigation frame likely accounts for the wide-ranging (0–46.4%) prevalence of IE described in a retrospective cohort study and systematic review that focused only on *S. lugdunensis* species and BSI [19].

In recent years, a growing number of studies have reported an increase in the occurrence of CoNS IE. The reason for this observation may be explained in part due to an aging population in developed countries and an increase in invasive cardiac procedures (i.e., placement of valve prostheses including TAVR and other types of cardiovascular devices [5,18,20,21]). The results of our investigation support the notion that older age, presence of multiple comorbid conditions, and indwelling cardiovascular devices are factors associated with the risk of definite IE due to CoNS. Indeed, 41.1% of those who had CoNS BSI and CIED developed definite IE, as did 48.8% of those with a prosthetic valve. The multivariable analysis confirmed an increased risk of definite IE in patients with foreign devices in the setting of CoNS BSI and support the hypothesis that some of these CoNS species have unique microbiological characteristics that support the initial attachment and their survival on the surface of foreign devices. CoNS IE was mostly associated with cardiovascular devices, chronic hemodialysis, CIED in another study [22]. In line with that, the Duke-ISCVID Working Group added clinical features to the list of possible minor criteria as predisposing conditions, including additional types of cardiac prosthetic material (e.g., transcatheter valve implant/repair and endovascular leads of CIEDs) [15].

CoNS as a cause of IE is well-recognized in the setting of prosthetic valves and is being increasingly observed as a cause of NVE [22,23,24,25,26]. In the current investigation, 23 (46.9%) of 49 definite IE cases involved native valves. In 8 (34.8%) of them no known predisposing cardiac conditions or CIED was present; 6 of these 8 IE cases were due to *S. epidermidis* and 2 due to *S. lugdunensis*. The course and outcome of CoNS NVE may have been underestimated in the past. Published studies show that these cases are complicated with high rates of valvular destruction, heart failure, and death [22,24,25,26,27]. Similarly, in our study, NVE cases had high rates of morbidity and mortality, with 60.9% developing complications and a 30.4% in-hospital mortality. The most common complications in patients with definite IE were perivalvular abscess (28.6%) and central nervous system emboli (22.4%). In the entire cohort, 47.2% of patients with definite IE required ICU admission, and 34.7% required surgery for definitive management. Early mortality rates were high, with 18.4% in-hospital mortality and 30.6% six-month mortality. Taken together, our data and that of others indicate that both CoNS NVE and PVE is associated with a considerably high complication rate and poor outcome. The increasing prevalence of CoNS BSI together with that of IE prompts a reconsideration of CoNS as serious reemerging pathogens and underscores the value of this study.

It may be reasonable to expect that TTP is shorter in definite IE in comparison to possible IE/BSI only, though, to our knowledge, there is no evidence that supports this notion. Patients with possible IE/BSI only had a shorter TTP but note that there was a considerable overlap of the IQR between the two groups (median 19 [IQR 15–22] vs. 21 [IQR 16–24] hours). Although, patients with possible IE/BSI only were more likely to have a central or peripherally inserted central catheter (54.5% vs. 32.7%, *p* = 0.009), still approximately 1/3 of the IE cases had a foreign body material to which CoNS bacteremia may have seeded. In these cases, the presence of both a colonized catheter and definite IE is possible, which in return may end in a shorter TTP in the blood culture obtained from the colonized central line. Moreover, as with all unadjusted analyses, this unadjusted association describing the marginal relationship between TTP and definite IE was susceptible to confounding bias and should be interpreted cautiously.

Our study has limitations. The nature of a retrospective observational investigation has inherent biases. The relatively high rate (19.8%) of definite IE among patients who underwent echocardiography was likely reflective of a selection bias of patients with clinical features that prompted clinicians to conduct further investigation for IE. This is much higher than that (8.1%) of patients described in a nationwide evaluation [18] who had at least two blood cultures positive for CoNS and had IE in a sensitivity analysis. Also, we focused only on patients who had undergone echocardiography ≤ 1 week prior to and ≤4 weeks after the first positive blood culture, because the investigations for IE (i.e., blood cultures and echocardiography) are typically performed in a close time frame. While blood cultures are frequently obtained prior to echocardiography, our approach did not exclude episodes in which echocardiography was performed shortly prior to the date of obtaining BC or BC positivity. However, by only including these episodes, we possibly introduced a bias with the exclusion of BSI patients who did not undergo echocardiography. Considering the high complication rate and poor outcome of CoNS IE, it is unlikely that we would have missed a significant number of IE cases in the 6.5-year study period. We did not collect data regarding indications for echocardiography and types of echocardiography done in the possible IE/BSI only group. Whether a TTE or a TEE was done is important as we consider the enhanced sensitivity of TEE to detect IE as compared to that of TTE and the ordering clinicians’ level of concern for complicating IE.

## 5. Conclusions

The impact of CoNS species designation on the rate of definite IE was identified in a subset of BSI patients who had undergone echocardiography and patients with *S. lugdunensis* BSI. These findings support the notion that species designation in CoNS BSI may be useful in diagnostic strategies to identify definite IE in patients with CoNS BSI.

## Figures and Tables

**Table 1:** Baseline Demographics and Clinical Characteristics of Patients with Definite IE and Patients with Possible IE/BSI Only.

Characteristic	Overall (*n* = 247)	Definite IE (*n* = 49)	Possible IE/BSI Only (*n* = 198)	*p* Value
Male	165 (66.8%)	35 (71.4%)	130 (65.7%)	0.442 ^1^
Age, years	64.6 (53.8–72.6)	69.5 (59.7–75.7)	62.8 (53.0–70.8)	0.017 ^2^
Race				0.918 ^1^
White	224 (90.7%)	45 (91.8%)	179 (90.4%)	
Black/African American	10 (4.0%)	2 (4.1%)	8 (4.0%)	
Other	13 (5.3%)	2 (4.1%)	11 (5.6%)	
Category of CoNS BSI				<0.001 ^1^
Community-acquired	32 (13.0%)	12 (24.5%)	20 (10.1%)	
Healthcare-associated	123 (49.8%)	30 (61.2%)	93 (47.0%)	
Nosocomial	92 (37.2%)	7 (14.3%)	85 (42.9%)	
Charlson Comorbidity Index				
Severity-weighted score	2 (0–7)	3 (1–8)	2 (0–7)	0.196 ^2^
Age- and severity-weighted score	5 (2–9)	5 (3–10)	4 (2–9)	0.085 ^2^
Diabetes mellitus	63 (25.5%)	17 (34.7%)	46 (23.2%)	0.099 ^1^
Moderate/severe renal disease	65 (26.3%)	17 (34.7%)	48 (24.2%)	0.137 ^1^
Chronic heart failure	74 (30.0%)	26 (53.1%)	48 (24.2%)	<0.001 ^1^
Chronic pulmonary disease	70 (28.3%)	13 (26.5%)	57 (28.8%)	0.754 ^1^
Other type of cancer	71 (28.7%)	9 (18.4%)	62 (31.3%)	0.073 ^1^
Metastatic solid tumor	13 (5.3%)	2 (4.1%)	11 (5.6%)	0.679 ^1^
Injection drug use	2 (0.8%)	1 (2.0%)	1 (0.5%)	0.283 ^1^
Hemodialysis	63 (25.5%)	11 (22.4%)	52 (26.3%)	0.583 ^1^
Catheter	56 (88.9%)	9 (81.8%)	47 (90.4%)	
Fistula	1 (1.6%)	1 (9.1%)	0 (0.0%)	
Graft	6 (9.5%)	1 (9.1%)	5 (9.6%)	
Immunosuppressive therapy	73 (29.6%)	11 (22.4%)	62 (31.3%)	0.223 ^1^
Valve disease	83 (33.6%)	32 (65.3%)	51 (25.8%)	<0.001 ^1^
Prosthetic valve	41 (16.6%)	20 (40.8%)	21 (10.6%)	<0.001 ^1^
CIED	56 (22.7%)	23 (46.9%)	33 (16.7%)	<0.001 ^1^
PICC/central line	122 (49.4%)	16 (32.7%)	106 (53.5%)	0.009 ^1^
Vascular graft/stent	27 (10.9%)	6 (12.2%)	21 (10.6%)	0.742 ^1^
Prosthetic joint	33 (13.4%)	13 (26.5%)	20 (10.1%)	0.002 ^1^
Neurologic device	1 (0.4%)	0 (0.0%)	1 (0.5%)	0.618 ^1^

Values represent frequency (percentage) for discrete variables and median (quartile 1 to quartile 3) for continuous variables. *p* values are by ^1^ Pearson χ^2^ or ^2^ Wilcoxon rank sum tests. BSI, bloodstream infection; CCI, Charlson comorbidity index; CIED, cardiovascular implantable electronic device; CKD, chronic kidney disease; COPD, Chronic obstructive pulmonary disease; IE, infective endocarditis; IV, intravenous; LVAD, left ventricular assist device; PICC, peripherally inserted central catheter.

**Table 2:** CoNS Species Distribution in Patients with Definite IE and Patients with Possible IE/BSI Only, and Comparison of Time to Positivity.

Characteristic	Overall (*n* = 247)	Definite IE (*n* = 49)	Possible IE/BSI Only (*n* = 198)	*p* Value
CoNS species				<0.001 ^1^
*S. epidermidis*	196 (79.4%)	33 (67.3%)	163 (82.3%)	
*S. lugdunensis*	22 (8.9%)	13 (26.5%)	9 (4.5%)	
*S. devriesei/haemolyticus*	11 (4.5%)	1 (2.0%)	10 (5.1%)	
*S. capitis*	7 (2.8%)	1 (2.0%)	6 (3.0%)	
*S. hominis*	6 (2.4%)	0 (0.0%)	6 (3.0%)	
Other CoNS *	5 (2.0%)	1 (2.0%)	4 (2.0%)	
Methicillin resistance	163 (66.5%)	25 (52.1%)	138 (70.1%)	0.018 ^1^
Time to positivity, hours	19.0 (15.0–22.5)	21.0 (16.0–24.0)	19.0 (15.0–22.0)	0.035 ^2^

Values represent frequency (percentage) for discrete variables and median (quartile 1 to quartile 3) for continuous variables. *p* values are by ^1^ Pearson χ^2^ or ^2^ Wilcoxon rank sum tests. * Infrequent CoNS species were grouped into a single ‘other’ level, which *includes S. capitis* (*n* = 1), *S. caprae* (*n* = 1), *S. pettenkoferi* (*n* = 1), *S. simulans* (*n* = 1), and *S. warneri* (*n* = 1). BSI, bloodstream infection; IE, infective endocarditis.

**Table 3:** Multivariable Analysis of Risk Factors Associated with Definite IE.

		Odds Ratio (95% Confidence Interval)	*p* Value
**Model 1**			
Age	(per 10 years)	1.18 (0.92–1.52)	0.196
Charlson Comorbidity Index	(per 1 point)	0.98 (0.89–1.08)	0.681
Hemodialysis		0.99 (0.41–2.40)	0.980
Valve disease		3.24 (1.54–6.80)	0.002
Foreign device		6.28 (2.98–13.26)	<0.001
**Model 2**			
Charlson Comorbidity Index *	(per 1 point)	0.98 (0.90–1.06)	0.585
Hemodialysis		1.27 (0.52–3.12)	0.595
Valve disease		3.27 (1.51–7.07)	0.003
Foreign device		6.51 (2.98–14.21)	<0.001
CoNS Species			0.002
*S. lugdunensis*	vs. *S. epidermidis*	8.02 (2.40–26.90)	
*S. lugdunensis*	vs. other CoNS species	12.89 (2.34–70.90)	

Multivariable analysis using logistic regression was performed to assess the risk of definite IE from pre-selected covariates. Two models were fitted, one that excluded CoNS species as a covariate (Model 1) and one that included species (Model 2). Foreign device indicates whether a patient had either a CIED or prosthetic valve at the time of BSI. * Due to sample size constraints and the addition of terms for species to Model 2, the effects for age and CCI were captured into a single covariate using the age-weighted CCI score.

## Data Availability

Data available upon request.
